# Atorvastatin-Induced Liver Injury With Concurrent Rhabdomyolysis After a Positive Rechallenge

**DOI:** 10.14309/crj.0000000000001570

**Published:** 2024-12-30

**Authors:** Harjit Singh, Adam Khalaf, Bryce F. Kunkle, Sherry Gholami, James H. Lewis, Amol S. Rangnekar

**Affiliations:** 1 Department of Internal Medicine, MedStar Georgetown University Hospital, Washington, DC; 2 Department of Gastroenterology and Hepatology, Medstar Georgetown University Hospital, Washington, DC; 3 Medstar Transplant Institute, MedStar Georgetown University Hospital, Washington, DC

**Keywords:** DILI, drug-induced liver injury, atorvastatin-induced liver injury, rhabdomyolysis, positive rechallenge

## Abstract

Statin-induced liver injury has been widely described. However, cases of clinically significant liver injury are rare. We present a 56-year-old woman who developed atorvastatin-induced grade III acute liver injury with concurrent rhabdomyolysis that worsened after rechallenging, which highlighted the need for pharmacovigilance with statins. The Roussel Uclaf Causality Assessment Method and Revised Electronic Causality Assessment Method both showed that atorvastatin was highly probable in causing hepatotoxicity. To our knowledge, this is the first reported case of concurrent drug-induced liver injury and rhabdomyolysis after a positive rechallenge with atorvastatin.

## INTRODUCTION

Statins are commonly used for primary and secondary prevention of cerebrovascular accidents (CVAs) and cardiovascular events.^1^ Although statins are generally well tolerated, adverse events, such as rhabdomyolysis, and idiosyncratic drug-induced liver injury (DILI) have been well described.^2^ The presentation of statin-related DILI varies, ranging from cholestatic hepatitis to mixed liver injury and frank hepatocellular injury.^3^ The incidence of statin-induced liver injury is estimated to be up to 3% in patients with the majority of cases demonstrating only minor elevations in liver enzymes that often resolve spontaneously.^4^ The overall incidence rate for statin-induced acute liver failure (ALF) has been reported to be between 1 and 2 per 100,000 person-years.^2,5^ This is close to the baseline level for ALF. Russo et al analyzed 1,188 cases of clinically significant DILI enrolled in the US Drug-Induced Liver Injury Network between 2004 and 2012, and 22 were attributed to statins.^4^ Of these 22 patients, 9 were hospitalized, and 4 developed ALF with 1 subsequent death. While the US Drug-Induced Liver Injury Network does not address incidence rates, the number of cases relative to the number of prescriptions is low. Although the risk of serious hepatic adverse events is low, pharmacovigilance around statin-induced DILI is important. We report a case of a 56-year-old woman who developed atorvastatin-induced grade III acute liver injury (ALI) complicated by rhabdomyolysis and acute renal failure requiring hemodialysis, with recurrence of liver injury on rechallenge.

## CASE REPORT

A 56-year-old woman with a history of hypertension and ischemic CVA and no history of liver disease presented as a transfer to our liver transplant center for the management of ALI. She presented with a 1-week history of new-onset confusion, jaundice, myalgias, and dark urine. On admission, her laboratory results were notable for an alanine aminotransferase 541, aspartate aminotransferase (AST) 1179, alkaline phosphatase (ALP) 383, total bilirubin 15.8, direct bilirubin 11.8, international normalized ratio 1.3, platelets 70, and creatine kinase (CK) 1760. R-factor was 4.2 indicating mixed liver injury. Initial workup was negative for viral, autoimmune, vascular, and other drug toxicity as potential etiologies of ALI. Of note, she was initiated on atorvastatin 40 mg, clopidogrel 75 mg, and aspirin 81 mg for a CVA that occurred 4 months before presentation, and her liver enzymes were normal at that time. All 3 of these medications were held on admission due to ALI and concern for developing ALF. She was not on other new medications or herbal supplements in the year before and did not use alcohol or illicit substances. Subsequently, magnetic resonance imaging brain demonstrated multiple scattered acute infarcts with concern for a cardioembolic source, and an echocardiogram with bubble revealed a patent foramen ovale. Intravenous N-acetylcysteine was started, and her liver enzymes and function improved over the course of 3 days with laboratory values improving to ALT 406, AST 792, INR 0.9, total bilirubin 3.1, and ALP 259.

**Figure 1.**
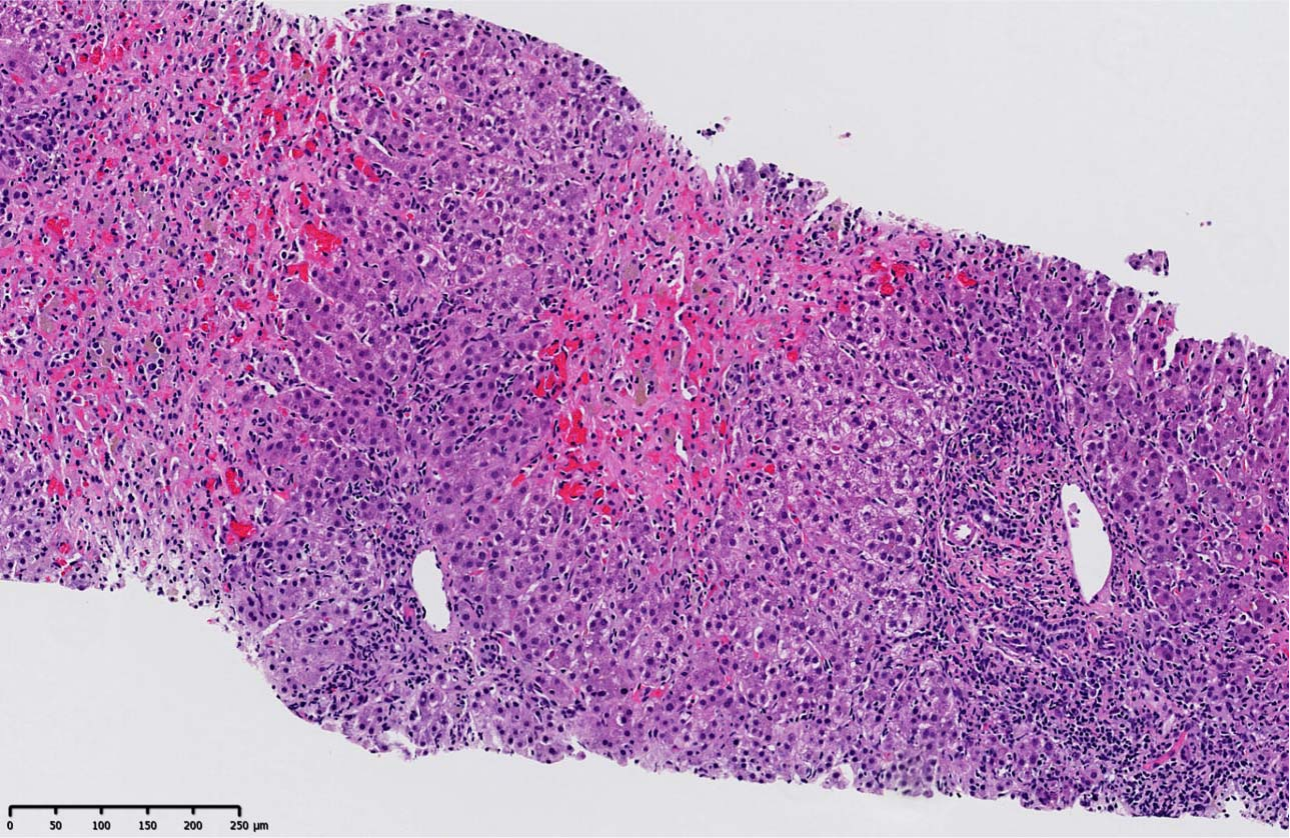
Liver biopsy demonstrating severe, acute neuroinflammatory hepatitis with zone 3 necrosis and an eosinophil predominant infiltrate with some plasma cells concerning for drug-induced liver injury.

Given the new CVA, she was rechallenged with atorvastatin 40 mg. Her liver enzymes began to rise 5 days after statin reintroduction, and the patient redeveloped worsening hepatocellular injury. Intravenous N-acetylcysteine was restarted. Her liver enzymes peaked at an ALT 1001, AST 4169, and ALP 623. R-factor was 4.8 indicating a similar mixed liver injury pattern. To rule out other causes of ALI, liver biopsy was done and demonstrated severe, acute neuroinflammatory hepatitis with zone 3 necrosis and an eosinophil predominant infiltrate with some plasma cells (Figure 1). These findings were very suggestive of DILI as the cause of ALI. Simultaneously, she developed severe rhabdomyolysis with a peak CK level of 316,454 IU complicated by acute renal failure requiring hemodialysis following the rechallenge. Atorvastatin was permanently discontinued, and her liver enzymes, renal function, and CK levels returned to normal with supportive management over the course of 3 months.

## DISCUSSION

DILI is one of the leading causes of ALF and ALI worldwide.^6,7^ Our case highlights a rare presentation of atorvastatin-induced grade III ALI with rhabdomyolysis that worsened after rechallenging with the same dose of atorvastatin. Severe rhabdomyolysis is associated with an elevation in both AST and ALT; however, coagulation markers and bilirubin levels can be used to differentiate ALI from rhabdomyolysis.^8^ In our patient, an elevated international normalized ratio (INR), total bilirubin, ALT, and AST with only a mild elevation in CK on initial presentation provided evidence of true ALI. Although CK was markedly elevated with the rechallenge of atorvastatin, simultaneous liver biopsy demonstrated evidence of DILI. These findings suggested the presence of concurrent statin-induced DILI and severe rhabdomyolysis due to a clinically positive rechallenge of atorvastatin.

Of the statins currently available, the risk of DILI appears to be highest with atorvastatin based on data collected from the US DILI Network prospective study and from the US Food and Drug Administration (FDA) Adverse Event Reporting System database.^4,7^ Atorvastatin was implicated in 8 of 22 (36.6%) patients with statin-related DILI in the US DILI Network study and was found to the be the fifth highest drug associated with DILI among all drug categories in the US FDA Adverse Event Reporting System database from 1997 to 2019.^4,7^ However, the incidence of statin-related DILI remains low at 1.9% based on the US DILI Network study.^4^ Given the large number of patients taking statins, they remain quite safe overall. The FDA has eliminated the need for liver test monitoring in patients with normal baseline values such as in our patient. In addition, statins are increasingly being used in patients with cirrhosis to reduce the risk of decompensation.^9^

When evaluating cases of DILI, causality assessments such as the updated Roussel Uclaf Causality Assessment Method score and the newer digitalized Revised Electronic Causality Assessment Method (RECAM) score are helpful as there are no reliable biomarkers to aid in diagnosis.^10–13^ In this case, a Roussel Uclaf Causality Assessment Method score of 9 (Table 1) suggested atorvastatin as a highly probable cause of ALI, and a RECAM score of 14 (Table 2) also provided evidence for atorvastatin as a highly probable cause of DILI.

**Table 1.** RUCAM Scoring for Atorvastatin using Cholestatic/Mixed Liver Injury Subscale

RUCAM criteria	Case scoring details	Score
Time to onset/Latency	>90 d	+1
Clinical course after drug withdrawal	Decrease in ALP >50% within 180 d	+2
DILI risk factors	Age >55 y	+1
Concomitant use of hepatotoxic drugs/herbs	None	0
Exclusion of other causes of liver injury	All causes in group I and II ruled out	+2
Previous hepatotoxicity of the drug	Reaction labeled in the product characteristics	+2
Response to readministration	Doubling of ALP with other drugs initiated at the same time	+1
Total RUCAM Score	9	

ALP, alkaline phosphatase; DILI, drug-induced liver injury; RECAM, Revised Electronic Causality Assessment Method.

**Table 2.** RECAM scoring for atorvastatin for mixed liver injury

RECAM domains	Case scoring details	Score
Domain 1A & 1B	Onset >90 d after drug start and onset while taking drug	0
Domain 2: Dechallenge	Initial R-value <5 and both ALP and total bilirubin declined to less than 50% of peak between 1 and 30 days after discontinuation	4
Domain 3: LiverTox category	Likelihood score of A (well-known cause of clinically apparent liver injury)	3
Domain 4: Exclusion of competing diagnoses	All other categories ruled out	0
Domain 5: Additional data	Re-exposure resulted in same R-value category with ALP increase > 2 × baseline and a latency period <60 d Liver biopsy with features of DILI	7
Total RECAM Score	14	

ALP, alkaline phosphatase; DILI, drug-induced liver injury; RECAM, Revised Electronic Causality Assessment Method.

Risk factors for statin-associated DILI and rhabdomyolysis include a variety of factors, including genetic predispositions and drug interactions. Variants in the SLCO1B1 gene, which encodes a transporter protein involved in statin metabolism, have been linked to elevated statin levels in the blood, raising the risk of both DILI and rhabdomyolysis.^14^ In addition, polymorphisms in various cytochrome P450 (CYP450) enzymes and specific human leukocyte antigen alleles involved in statin metabolism may contribute to an increased risk of liver injury and rhabdomyolysis.^14–16^ Coadministration of other drugs that are metabolized through the CYP450 pathway such as certain antifungals, human immunodeficiency virus protease inhibitors, fibrates, and macrolide antibiotics increases the risk of both DILI and rhabdomyolysis due to altered statin metabolism.^17,18^ Therefore, it is crucial to exercise heightened vigilance when prescribing these medications to individuals with a history of statin use.

To our knowledge, this is the first reported case of concurrent DILI and rhabdomyolysis after a positive rechallenge with atorvastatin and demonstrated the continued need for pharmacovigilance in statin-related adverse effects.

## DISCLOSURES

**Author contributions:** H. Singh participated in writing, editing, organizing the manuscript, making the tables, and collecting the images. A. Khalaf, BF Kunkle, and S. Gholami participated in writing and editing the manuscript. JH Lewis and AS Rangnekar participated in the editing and revisions for the manuscript. H. Singh is the article guarantor.

**Financial disclosure:** None to report.

**Previous presentation:** This case was presented as a poster presentation at the American College of Gastroenterology Annual Scientific Meeting; October 20–25, 2023; Vancouver, BC, Canada.

Informed consent was obtained for this case report.
